# Good Cop, Bad Cop: The Opposing Effects of Macrophage Activation State on Maintaining or Damaging Functional β-Cell Mass

**DOI:** 10.3390/metabo10120485

**Published:** 2020-11-26

**Authors:** Daelin M. Jensen, Kyle V. Hendricks, Austin T. Mason, Jeffery S. Tessem

**Affiliations:** Nutrition, Dietetics and Food Science Department, Brigham Young University, Provo, UT 84602, USA; daelinmichael@gmail.com (D.M.J.); kylev.hendricks@gmail.com (K.V.H.); austintaylormason@gmail.com (A.T.M.)

**Keywords:** β-cell, macrophage, islet, cytokine, diabetes

## Abstract

Loss of functional β-cell mass is a hallmark of Type 1 and Type 2 Diabetes. Macrophages play an integral role in the maintenance or destruction of pancreatic β-cells. The effect of the macrophage β-cell interaction is dependent on the activation state of the macrophage. Macrophages can be activated across a spectrum, from pro-inflammatory to anti-inflammatory and tissue remodeling. The factors secreted by these differentially activated macrophages and their effect on β-cells define the effect on functional β-cell mass. In this review, the spectrum of macrophage activation is discussed, as are the positive and negative effects on β-cell survival, expansion, and function as well as the defined factors released from macrophages that impinge on functional β-cell mass.

## 1. Introduction

The prevalence of diabetes is growing. It is currently estimated that 463 million individuals are diabetic and that by the year 2045 that number will increase to 700 million [1]. While the etiologies of the two primary forms of diabetes are clearly different, Type 1 Diabetes (T1D) and Type 2 Diabetes (T2D) both result in decreased functional β-cell mass (defined as changes in β-cell survival, proliferation, and insulin secretion). T1D is characterized by autoimmune destruction of the insulin-producing pancreatic β-cells [2], and T2D is characterized by β-cell dysfunction and the ultimate loss of β-cell maturity and increased β-cell death [3].

While clearly observed with T1D, the increased presence and islet infiltration of hematopoietic cells are also observed in the pancreas of T2D patients [4,5]. Additionally, resident monocyte-derived dendritic cells and macrophages also play a critical role in β-cell homeostasis [6]. Signaling from these cell types can result in modifications in β-cell function, survival, and proliferation. The direct interaction between hematopoietic cells and β-cells plays a critical role in the maintenance of functional β-cell mass.

Resident macrophages are found in all human tissues. The entire macrophage pool in an adult human is estimated to be about 10^10^ cells [7]. Macrophages are a critical part of the innate immune response that specializes in the detection and destruction of foreign pathogens as well as the activation and recruitment of adaptive immune cells. Inflammatory macrophages have classically been considered to be detrimental to β-cell function and survival, thereby contributing to β-cell failure in both T1D and T2D. Recent findings, however, have demonstrated that anti-inflammatory macrophages play a supportive role through tissue remodeling that protects β-cells and enhances insulin secretion and replication. These contradictory effects of the macrophage on the β-cell are due to the macrophage activation state and the factors that are produced by and released from macrophages found in the pancreatic islet. In this review, the deleterious and protective effects of macrophages on the β-cell are described in the context of macrophage activation states and the factors secreted by macrophages that signal to the β-cell. Further understanding of the origins and activation pathways of tissue-resident macrophages is fundamental for the design of intervention strategies to maintain functional β-cell mass as a treatment for T1D and T2D.

## 2. The Macrophage Activation Spectrum

Macrophages play an important role in maintaining tissue homeostasis, completing essential tissue-specific functions, and protecting the organism from infection. Due to the presence of scavenger receptors, they are able to perform housekeeping tasks such as removal of aged red blood cells, necrotic tissue, and toxic molecules, in the absence of special activation-associated stimuli. However, under the distress of infected or injured tissue, these homeostatic functions are increased by a variety of activating stimuli [8].

Tissue-resident macrophages were thought to continuously repopulate from circulating monocytes, which are ontologically derived from hematopoietic stem cells [9]. Recent studies have challenged this view. Although monocytes have the ability to differentiate into macrophages, subpopulations of resident macrophages in certain tissues (such as the pancreas) result from yolk-sac derived precursors during embryonic development [10,11]. This suggests that the pancreatic macrophage population is able to be maintained independently of circulating monocytes [12].

**Figure 1:**
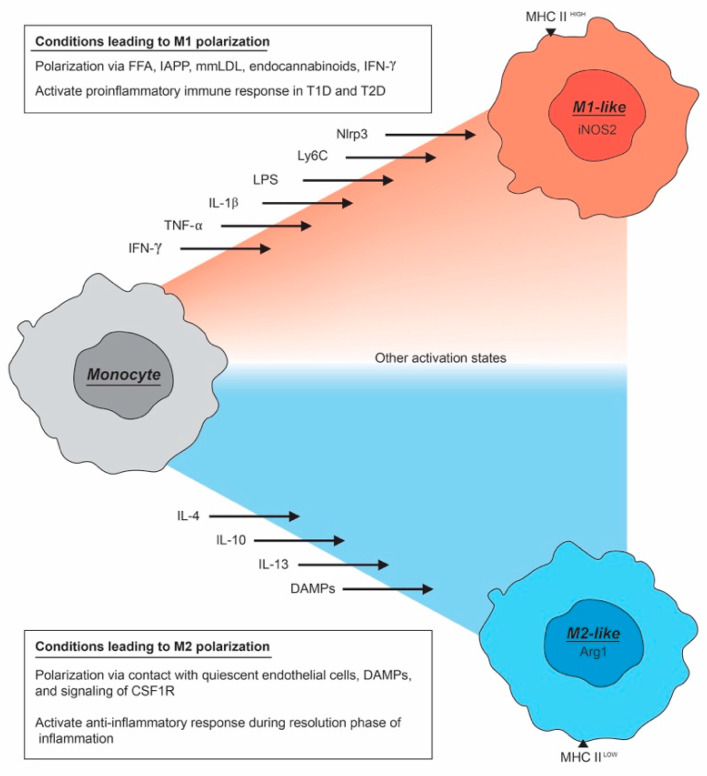
Polarization of monocytes to M1-like or M2-like macrophages. Macrophages can be polarized along an activation spectrum in response to different signals within their microenvironment. An undifferentiated macrophage will lean more M1-like under proinflammatory conditions, propagating a “kill” response. Conversely, a macrophage may lean more M2-like under anti-inflammatory conditions usually following M1 damage where a “heal” response is necessary.

Macrophages are traditionally divided into two functional subgroups; the classically activated, inflammatory and cytotoxic M1-like macrophages and the alternatively activated M2-like macrophages that are anti-inflammatory and mediate tissue repair and remodeling [10]. It is now understood that these subsets better represent different points on a spectrum of macrophage activation states [13] and that other activation states may well be present [14]. Macrophages are able to respond to specific environmental signals to express various activation states along a dynamic range of phenotypes [15]. Nevertheless, for the sake of simplicity, we will use the subgroups M1-like and M2-like macrophage designations (Figure 1).

M1-like macrophages are characterized by their immunogenic properties. These properties protect against infection by propagating proinflammatory responses [16]. In the case of obesity-related pancreatic inflammation that is common in T2D, an abundance of free fatty acids (FFA), reactive oxygen species (ROS), islet amyloid polypeptide (IAPP), and proinflammatory cytokines increase M1-like macrophage polarization [17]. An M1-like macrophage can further propagate this response through the activation of the non-obese diabetic (NOD)-, LRR- and pyrin domain containing 3 (NLRP3) inflammasome. Once activated, the NLRP3 inflammasome secretes cytokines such as interleukin-1 beta (IL-1β) that induce M1-like polarization of other macrophages [18]. The proinflammatory M1-like state is also enhanced in the presence of interferon-gamma (IFN-γ), lipopolysaccharide (LPS), IL-1β, and tumor necrosis factor-alpha (TNFα). Additionally, M1-like macrophages are efficient producers of effector molecules such as ROS and reactive nitrogen species (NOS). They also produce inflammatory cytokines, such as IL-1β, IL-6, IL-12, and TNF-α, and chemokines such as chemokine (C-X-C motif) ligand 9 (CXCL9), CXCL10, and Chemokine (C-C motif) ligand 5 (CCL5). Additionally, M1-like macrophages express major histocompatibility complex (MHC) class II. These factors allow the M1-like macrophage to be recognized by and induce an immune response from T helper 1 (Th1) cells [19].

The M2-like macrophage primarily serves an anti-inflammatory and tissue regenerative purpose. M2-like macrophage polarization has a wider variety of activating stimuli. Anti-inflammatory signals such as IL-4, IL-13, macrophage colony-stimulating factor (CSF-1), and transforming growth factor beta (TGF-β) are associated with differentiation to the M2-like tissue regenerative and remodeling phenotype [20]. IL-4 and IL-13 were among the first to be discovered as inhibitors of the classical M1-like phenotype [16]. IL-21 is another important cytokine that drives M2-like polarization. Additionally, IL-33 is a cytokine of the IL-1 family that amplifies IL-13-induced polarization of macrophages to the M2-like phenotype. The M2-like phenotypes are characterized by the expression of chitinase 3 like 1 (CHI3L1), CCL24, CCL17, and arginase 1. M1-like macrophages metabolize arginine through NOS2 to produce nitric oxide (NO), M2-like macrophages use arginase 1 (ARG1) to metabolize arginine into polyamines that are used for proliferation and tissue remodeling [16]. M2-like macrophages express Th2 attracting chemokines CCL17 and CCL24, allowing the M2-like macrophages to work synergistically with Th2 cells to resolve inflammation and promote tissue remodeling, angiogenesis, and immunoregulation. Even within the M2-like “classification” there appear to be M2-like subtypes based on in vitro activation studies, expression profile, and function. M2a macrophages are activated by IL-4 and IL-13, express scavenger and phagocytic receptors, and secrete fibronectin, insulin-like growth factor (IGF), and TGFβ. M2b macrophages produce the proinflammatory IL-1β, IL-6, and TNF-α, and the anti-inflammatory IL-10. M2b cells are activated by Toll-like receptors and IL-1 receptor antagonists. M2c macrophages are activated through IL-10 and glucocorticoids. They remove apoptotic cells through expression of tyrosine-protein kinase MER (MerTK). Finally, M2d macrophages express IL-10 and vascular endothelial growth factor (VEGF). This wide variety of “M2” macrophage subtypes emphasizes the current understanding that M1-like and M2-like macrophages define extremes of a “macrophage activation spectrum” in terms of activation, gene expression, and function [21,22,23,24].

## 3. Macrophages Can Impair β-Cell Function and Survival

Extensive findings demonstrate that M1-like macrophages have deleterious effects on β-cells by impairing glucose-stimulated insulin secretion (GSIS), inducing apoptosis, and causing β-cell dedifferentiation. NOD mice that spontaneously develop T1D, can have diabetes development impaired through macrophage depletion [25]. These findings have been substantiated in the BioBreeding Diabetes Prone rat (DP-BB) model [26]. Similarly, human tissue sections from T1D patients demonstrate macrophage infiltration of the islet [27,28]. The key proinflammatory cytokines observed in T1D are TNF-α, IL-1β, and IFN-γ, all of which are produced by macrophages [29]. Macrophages isolated from T1D patients have elevated proinflammatory gene expression. Interestingly, macrophages from long-term T1D patients have an impaired ability to undergo M2-like activation, suggesting potential changes to the macrophage population over time [30]. Macrophages exposed to oxidative stress produced elevated ROS levels that can push the macrophage toward an M1-like phenotype, which can be reverted to an M2-like phenotype with the use of ROS scavengers [31,32].

Islet inflammation is also a characteristic of T2D. Increased macrophage numbers have been observed in human T2D pancreata as well as rodent T2D models such as db/db mice, GK rats, and diet-induced obesity models [18,33,34,35,36,37,38,39]. Islets from T2D patients have elevated IL-6, IL-8, CXCL1, granulocyte colony-stimulating factor (GCSF), and macrophage inflammatory protein-1 alpha (MIP1α) levels [33]. Hyperglycemia and hyperlipidemia induce islet chemokine secretion which results in macrophage islet infiltration. These macrophages express M1-like markers, and depletion of M1-like macrophages suppresses β-cell lipotoxicity in vivo [9,36]. Various systemic changes associated with obesity can induce macrophage activation to an M1-like phenotype, and result in increased secretion of inflammatory cytokines such as IL-1β, IFN-γ, and TNF-α that directly signal to and affect β-cell function and survival [40]. Using clodronate-mediated macrophage suppression, db/db and KKAy mice have improved glucose tolerance and insulin secretion, demonstrating that macrophages in these T2D models impair β-cell function [36]. β-cell dedifferentiation is a hallmark of T2D and plays a major role in decreasing functional β-cell mass [41]. Db/db islets have impaired expression of β-cell identity genes, such as MAF BZIP Transcription Factor A (MafA), pancreatic and duodenal homeobox 1 (Pdx1), Glut2, and potassium inwardly rectifying channel subfamily J member 11 (Kcnj11). Interestingly, β-cell identity gene expression improves when macrophages are depleted from db/db islets [42]. These results demonstrate that M1-like macrophages have a deleterious effect on functional β-cell mass in T1D and T2D.

### Macrophage Produced Secreted Factors That Negatively Modulate Functional β-Cell Mass

The primary cytokines produced by macrophages that negatively impact functional β-cell mass are IL-1β, IL-6, IFN-γ, and TNF-α (Table 1). These cytokines have negative effects on functional β-cell mass by impairing GSIS, inducing cell death, and increasing β-cell dedifferentiation. IL-1β is a member of the interleukin 1 (IL-1) family of cytokines. As such, IL-1β is involved early in the immune response to signal the production of other cytokines. Although the islet is capable of producing IL-1β under diabetogenic conditions, mouse and human islet studies have shown that macrophages secrete the overwhelming majority of IL-1β [43]. Additionally, β-cells express high levels of interleukin-1 receptor (IL-1R), and as such are highly sensitive to IL-1β [44]. It has been shown that when β-cells are subject to acute exposure of IL-1β, GSIS and overall β-cell survival improve. However, numerous studies have shown that chronic exposure to IL-1β common to diabetogenic or obese conditions causes impaired GSIS and increased β-cell de-differentiation and death [43,45,46]. Blocking IL-1β or inhibiting IL-1R results in improved outcomes in the restoration of β-cell function, mass, and the reversal of T2D phenotypes [9,47,48].

IL-1β signaling results in activation of nuclear factor kappa-light-chain-enhancer of activated B cells (NF-κB) and mitogen-activated protein kinase (MAPK) pathways [49], through which the β-cell releases chemokines such as CXCL1 and CXCL2 to recruit cells of the innate and adaptive immune system. In a study using neonatal NOD mice, those treated with a neutralizing anti-IL-1β monoclonal antibody (mAb) had decreased macrophage and neutrophil islet infiltration [50]. Similarly, mice treated with anti-IL-1β mAb demonstrated a significant decrease in islet produced CXCL1 and CXCL2, which recruits infiltrating immune cells through the CXCR2 receptor [51,52]. The use of IL-1R antagonist drugs has shown significant reductions in islet macrophage infiltration [53]. Finally, β-cells exposed to IL-1β increase expression and secretion of other proinflammatory signals monocyte chemoattractant protein-1 (MCP-1), IL-6, and TNF-α, thus inducing greater macrophage migration and islet inflammation [18]. The combined exposure of islets to IL-1β and IL-6 decreases GSIS, increases endoplasmic reticulum (ER) stress marker expression (such as Inducible nitric oxide synthase 2 (iNOS2), activating transcription factor 4 (ATF4), and CCAAT/enhancer binding protein (C/EBP) homologous protein (CHOP)), induces calcium handling deficiencies, and increases cell death [54]. These data demonstrate the IL-1β signaling at the β-cell results in the production of signals to enhance islet inflammation.

Culturing macrophages ex vivo with elevated glucose or fatty acids induces IL-1β release [47,55]. Min6 β-cells cultured with conditioned media from palmitate-treated macrophages demonstrated that preclearing the media with neutralizing antibodies blocked the effect of these cytokines to impair β-cell function [36]. Macrophages cultured with Min6 β-cells result in increased cytokine secretion, which impedes GSIS, and the addition of anti-IL-1β and anti-TNF-α antibodies improves GSIS [36]. β-cell IL-1β signaling through the MAPK and c-Jun N-terminal kinase (JNK) pathway results in downregulation of the phosphatidylinositol 3-kinase-protein kinase B signaling pathway (P13K-AKT) signaling cascade, and ultimately decreases PDX1-mediated gene expression [45,56]. This results in a decrease in insulin mRNA levels and impaired GSIS [57,58,59,60,61,62,63].

IL-1β induces pro-apoptotic and necrotic pathways in the β-cell through the extracellular signal-regulated kinase (ERK) signaling pathways [64]. ER stress that is characteristic of T2D β-cells potentiates the IL-1β signaling pathway and leaves the β-cell more susceptible to IL-1β-mediated cell death [54]. TNF-α and IFN-γ work synergistically with IL-1β to induce β-cell apoptosis via the intrinsic and extrinsic apoptotic pathways. IL-1β and IFN-γ induce the intrinsic apoptotic pathway through the activation of the NF-κB-mediated gene network. NF-κB activation subsequently leads to NO and cytokine production, depletion of ER calcium stores, and induction of ER stress. Rat islets cultured with IL-1β and IFN-γ revealed expression changes to genes associated with inflammation, cell death, antigen presentation, and cytokines/chemokine production [65]. ER stress results in mitochondrial damage, cytochrome c release, and mitochondrial death signals that activate caspase 9 and caspase 3 resulting in activation of the intrinsic apoptosis pathway [66,67]. Human islets cultured with TNF-α, IFN-γ, and IL-1β with or without NO induced ER stress as indicated by increased expression of CHOP, activating transcription factor 3 (ATF3), binding immunoglobulin protein (BIP), and X-box binding protein-1 (XBP1). IL-1β can also induce apoptosis via the extrinsic pathway by up-regulating Fas receptor expression [68].

IL-1β can induce β-cell dedifferentiation. Human and rodent islets cultured with IL-1β, IL-6, and TNF-α present with β-cell dedifferentiation [69]. Furthermore, IL-1β induces downregulation of forkhead box protein O1 (Foxo1), which is essential for maintaining β-cell differentiation. Similar results are observed with EndoC-βH1 and human islets, where culture with the same mixture of cytokines induces upregulation of progenitor genes such as SRY-Box Transcription Factor 9 (Sox9), and downregulation of mature β-cell genes [70]. Interestingly, culturing β-cells with non-cytotoxic IL-1β levels impaired insulin secretion, reduced β-cell proliferation, and decreased expression of β-cell identity genes such as MafA and Urocortin-3 (Ucn3) [71]. These changes, however, were reversible as IL-1β removal restored β-cell identity gene expression. While these data strongly suggest that IL-1β plays a critical role in inducing β-cell dedifferentiation, pancreatic IL-1R deletion (the receptor by which IL-1β signal transduction occurs) also results in impaired β-cell function and increased expression of the dedifferentiation marker Aldh1a3 [45]. More studies will need to be completed to clearly understand the role of IL-1β on β-cell dedifferentiation.

One of the key differences between M1-like and M2-like macrophages is arginine metabolism. M1-like macrophages use arginine to produce NO by way of iNOS2. Macrophage production of NO and ROS have direct effects on functional β-cell mass due to low β-cell expression of radical scavenging pathways. NF-κB regulates the expression of inducible nitric oxide synthase (iNOS) in β-cells, with many of the gene expression changes associated with cytokine exposure being secondary to iNOS-mediated NO formation. Increased macrophage produced NO and ROS leads to DNA damage and activation of poly (ADP-ribose) polymerase (PARP) to facilitate DNA repair. PARP activation depletes the β-cells NAD pool, which can lead to β-cell necrosis [72]. IL-1β stimulates β-cell iNOS expression, which leads to elevated internal NO levels. Increased cellular NO impairs electron transfer, decreases mitochondrial ATP production, and induces the expression of proinflammatory genes in the β-cell [73].

IL-6 signals through the IL-6 receptor system. This results in signal transducer and activator of transcription 3 (STAT3) activation and MAPK activation and increased transcription of downstream target genes [74,75]. IL-6 is sufficient to impair GSIS and decrease islet insulin content [76,77]. Conversely, chemically inhibiting IL-6 signaling at the IL-6 receptor improves insulin content and GSIS [78]. The observed decrease in insulin content due to IL-6 is due to transcriptional changes, as IL-6 decreases Ins1, Ins2, and PDX1 mRNA levels [79]. IL-6 signaling may also impair mitochondrial function through inducing mitochondrial fission and potentially increased mitophagy [80].

IFN-γ is a pro-inflammatory cytokine responsible for β-cell destruction. IFN-γ signals to the β-cell through the interferon-gamma receptor (IFNGR) complex. This receptor complex activates the JAK/STAT and the NF-κB signaling cascades [49,81]. This leads to activation of the transcription factor interferon regulatory factor 1 (IRF-1) and upregulation of caspase-1, caspase-3, caspase-9 [82], and other proapoptotic gene expression [82]. Loss of STAT1 signaling impairs IFN-γ-mediated cell death [83]. Furthermore, loss of STAT1 impairs IP-10 and iNOS gene expression [83]. IFN-γ also impairs β-cells insulin content and GSIS [84,85]. Finally, IFN-γ potentiates IL-1β-mediated iNOS expression and NO production in the β-cell [86,87,88].

TNF-α was originally thought to be produced by T-cells, however, data demonstrate that TNF-α is primarily produced by macrophages and dendritic cells in the islet [89,90]. TNF-α signals through the TNFR and activates the NF-κB and MAPK signaling cascades [49,91]. Once a threshold of activation through NF-κB is activated, proapoptotic and inflammatory pathways are activated [92]. Inhibition of TNF-α, NF-κB, and JNK has been shown to improve β-cell survival and function [93]. These signaling cascades can lead to β-cell dysfunction and death [93]. TNF-α, IL-1β, and IFN-γ all induce ROS production in β-cells through activation of nicotinamide adenine dinucleotide phosphate (NADPH) oxidases [94]. They also upregulate iNOS expression, resulting in increased NO production [95,96]. Increased ROS and NO production results in mitochondrial damage and activation of the intrinsic apoptotic pathway [97]. Induction of iNOS through the TNF-α signaling cascade functions through the IRF transcription factor [98,99]. Human islets exposed to TNF-α, IL-1-β, and IFN-γ have increased expression of various cytokines that enhance immune cell migration, including CXCL1, CXCL8, CCL20, CCL2, and CXCL10 through the NF-κB and STAT1 signaling cascade [100]. TNF-α also induces β-cell Ca^2+^ influx, which can negatively affect insulin secretion and β-cell survival [101]. Finally, using a TNF-α antagonist partially restored β-cell identity gene expression that are lost during dedifferentiation [69]. These data demonstrate the negative effect that these various M1-like macrophages produced secreted factors have on the pancreatic β-cell.

## 4. M2-Like Macrophage Can Enhance the Development, Maintenance, and Function of β-Cells

There is an intimate relationship between macrophages and the development of pancreatic islets, and specifically β-cell proliferation and survival. Mature F4/80+ macrophages are found in the pancreatic bud by E14.5 [102]. The presence of these macrophages is directly linked to the expansion of β-cell number during development. Pancreas explants cultured with exogenous macrophage colony-stimulating factor (M-CSF), which is sufficient to induce macrophage proliferation, results in increased macrophage and β-cell number more than four-fold over the explants cultured without M-CSF, suggesting a connection between macrophage signaling and β-cell proliferation [102]. The presence and increased migration of macrophages to the developing pancreas is necessary for the delamination of endocrine cells from the pancreatic ducts and their ultimate migration to nascent islets [103]. Similarly, macrophages are observed in the human pancreas as early as 6 weeks of development, with elevated levels of the cytokine CSF-1 also being detected [104]. As CSF-1 is essential for macrophage differentiation, these data suggest that macrophages are needed for nascent β-cell expansion. CSF-1-deficient osteopetrotic CSF-1 op/op mice, which have impaired production of myeloid-derived macrophages and dendritic cells, also have decreased β-cell mass and impaired β-cell proliferation when compared to CSF-1 op/+ littermates [102,103]. These data demonstrate that macrophages are necessary for β-cell proliferation during embryonic development. Furthermore, given the function of these macrophages, these data suggest a M2-like phenotype.

While the data for macrophage-mediated β-cell proliferation during development are strong, there are equally compelling data regarding the effects of the macrophage on β-cell survival and proliferation during adulthood or during disease states. Mice fed a high-fat diet initially demonstrate increased β-cell proliferation. This proliferation correlates with increased intra-islet macrophage accumulation, where these macrophages express markers indicative of an M2-like activation state [105,106]. In fact, hyperplastic islets observed in diet-induced obesity have greater macrophage concentrations, suggesting that macrophages may be needed to open the extracellular matrix and create an expansion niche for the growing islet [33]. These studies are supported by other models that demonstrate the effect of macrophages on the β-cell. Macrophages with an M1-like expression profile shifted to an M2-like profile in response to diphtheria toxin-mediated β-cell damage and in correlation with the subsequent β-cell expansion [107,108]. Clodronate-mediated macrophage ablation attenuates β-cell regeneration, suggesting that macrophages are needed for β-cell expansion in this model. Similarly, pancreatic ductal ligation-mediated β-cell regeneration is dependent on M2-like macrophages [109,110,111,112]. Using a chronic pancreatitis model of β-cell loss, it was shown that M2-like activated macrophages were essential for β-cell proliferation and that transplantation with CSF-1R -/- bone marrow (which has impaired macrophage production) results in lost β-cell mass [113]. Changes in vascularization were also observed, and given the ability of macrophages to assist in vascular remodeling, some of the β-cell maintenance and proliferation may be vascular mediated [20]. β-cell regeneration after VEGF-A-mediated β-cell loss was shown to be dependent on M2-like macrophages [114,115]. Finally, recent observations demonstrate that human pancreatic donors demonstrated a strong correlation between the presence of M2-like macrophages and increased islet vascularization and β-cell proliferation marker expression [116]. These data demonstrate a direct connection between M2-like macrophages and the maintenance and expansion of β-cell mass in various models of β-cell damage and regeneration.

### Macrophage Produced Secreted Factors That Positively Modulate Functional β-Cell Mass

M2-like macrophages clearly play a protective role in β-cells in terms of maintaining and expanding functional β-cell mass. This protective role is mediated by various factors secreted from the macrophage that directly affect the β-cell (Table 2). Using a streptozotocin (STZ) model of β-cell destruction, recruitment of M2-like macrophage to islets was observed. It was shown that these M2-like macrophages produce and release wingless-type MMTV integration site family, member 3A (Wnt3a), which induces the Wnt/β-catenin pathway in the β-cell, which resulted in increased β-cell proliferation [117]. Similarly, using a diphtheria toxin-induced model of β-cell injury, M2-like macrophages were shown to induce β-cell survival and proliferation through macrophage produced Wnt and activation of the β-cell Wnt/β-catenin signaling pathway [107,118].

Resident macrophages can be moved to an M2-like phenotype through IL-33 signaling. In response to islet secreted IL-33 and the change of the macrophage phenotype to an M2-like state, changes in secreted factors are observed. This signaling cascade, also facilitated by IL-13 and CSF-2, induces expression of aldehyde dehydrogenase 1 family, member A2 (Aldh1a2), which produces retinoic acid. Retinoic acid released from resident macrophages (and dendritic cells) results in the induction of retinoic acid receptor beta (RARβ) expression in the β-cell and increased insulin [119].

Using a partial pancreatic ductal ligation demonstrates additional macrophage secreted factors that affect functional β-cell mass. M2-like macrophages were shown to migrate in response to ductal ligation and secrete TGFβ1 and EGF. TGFβ1 induced β-cell upregulation of mothers against decapentaplegic homolog 7 (SMAD7) and SMAD2, where SMAD7 is sufficient to induce proliferation and SMAD2 acts as an inhibitor of the SMAD7 proliferation cascade. In addition to secreting TGFβ1, the M2-like macrophages also secreted EGF. The EGF signaling cascade resulted in inhibition of SMAD2 nuclear localization. This allowed β-cells to undergo proliferation through upregulation of the cell cycle activators cyclin D1/D2 and nuclear exclusion of the cell cycle inhibitor p27^CDKN1B^ [111]. TGFβ has also been shown to be essential for proper pancreas development [120,121]. Furthermore, knockout of TGFβ receptor I and II in β-cells inhibits M2-like macrophages-mediated β-cell proliferation [111,121].

EGF is not the only growth factor secreted from macrophages that have been shown to have a positive effect on β-cells. Macrophages from obese mice have been shown to increase β-cell proliferation. This occurs due to PDGF release from the macrophage and signaling to the β-cell through platelet-derived growth factor receptor (PDGFR) [122]. Hepatocyte growth factor (HGF) has been shown to push macrophages to an M2-like phenotype [123], and M2-like macrophages are capable of secreting HGF [10]. HGF has been shown to increase β-cell proliferation and increase insulin secretion, as well as induce β-cell regeneration in a partial pancreatectomy model [124,125]. IGF-1 produced from M-2 like macrophages has been shown to play a critical role in maintaining functional β-cell mass by maintaining glucose-stimulated insulin secretion [126].

In addition to producing growth factors, M2-like macrophages release IL-10, which has direct effects on maintaining functional β-cell mass. β-cells exposed to IL-10 express elevated levels of anti-apoptotic genes [127]. IL-10 can induce β-cell iNOS protein levels, thus potentially decreasing NO levels [128]. Finally, IL-10 can enhance insulin secretion, as measured by c-peptide levels [129]. A recent publication showed that human islets have macrophages in the perivascular region that are the primary source of islet IL-10 levels, and that loss of the ability to produce IL-10 in the obese and diabetic state leads to β-cell loss [130].

Finally, islet expansion is dependent on the remodeling of the islet vasculature and the extracellular matrix (ECM), as well as the release of growth factors bound in the ECM. M2-like macrophages produce and secrete matrix metalloproteinases (MMP) that are critical for this process. Macrophages from human islets are the primary source of MMP9 [130]. Macrophages that migrate to the islets in a chronic pancreatitis model of diabetes express various M2-like markers, including MMP9, which is essential for islet vascularization and β-cell expansion [113]. Together, these data demonstrate the various factors secreted from the M2-like macrophages that have direct effects on maintaining functional β-cell mass.

## 5. Use of Macrophages to Improve Functional β-Cell Mass as a Treatment for Diabetes

The previous studies demonstrate the potential for M2-like macrophages to be harnessed to ameliorate functional β-cell mass as a treatment for T1D and T2D. Various studies have begun to demonstrate this feasibility. While macrophages from long-term T1D patients have impaired ability to undergo M2-like activation [30], NOD mice treated with the BET antagonist I-BET151 demonstrated significant M2-like activation and β-cell regeneration [131]. Similarly, NOD mice that received adoptive transfer of M2-like macrophages resulted in greater than 80% protection against T1D phenotypes for over 3 months compared to NOD mice that did not receive adoptive macrophage transfer or mice that received adoptive transfer of non-M2-like macrophages [132]. In this model, it was shown that the adoptively transferred M2-like macrophages preferentially migrated to the pancreas, where a greater number of islets were maintained after M2-like macrophage adoptive transfer. Using an STZ model of β-cell injury, it was shown that adoptive transfer of M2-like macrophages was sufficient to protect functional β-cell mass and diabetes-induced kidney damage. Mice that received M2-like macrophage adoptive transfer had greater islet area, as well as improved HbA1c and non-fasting blood glucose levels [133]. Finally, STZ mice transplanted with mesenchymal stem cells resulted in improved fasting blood glucose, β-cell area, and β-cell proliferation. This corresponded with increased infiltration of M2-like macrophages [117]. It was shown that the mesenchymal stem cells were inducing endogenous macrophages to undergo M2-like activation. The M2-like macrophages induced β-cell proliferation through induction of the Wnt3a/β-catenin signaling pathway. Although these preliminary rodent studies demonstrate the ability of M2-like macrophages to block or reverse diabetes progression and loss of functional β-cell mass, future studies in humans will be required.

## 6. Concluding Remarks

The purpose of this review was to highlight the detrimental and beneficial effects of macrophages, based on their activation state, on functional β-cell mass. From the studies reviewed here, it is clear that macrophages can have opposing effects on the β-cell. The various signals from the islet, from other autoimmune cells, and from systemic inflammation present in either T1D or T2D have the potential to induce M1-like activation of the macrophage and macrophage-mediated decrease in functional β-cell mass. Alternatively, signals from the β-cell under normal physiology or in response to certain forms of β-cell damage can result in M2-like macrophage activation and secretion of factors that promote β-cell proliferation, survival, and insulin secretion.

One potential caveat of these findings is the applicability to human β-cells. Given the physiological differences between rodent and human islets, it will be essential to validate the beneficial effects of M2-like macrophages that have been observed in rodent islets in human islets. Rodent islets or mice were the primary models used in the majority of studies cited in this review (Table 3). Fortunately, there are more and more studies looking at the effect of macrophages on human islets, or exploring the role of macrophages in vivo on human functional β-cell mass. For the greater application of these findings, these studies will need to be expanded.

**Figure 2:**
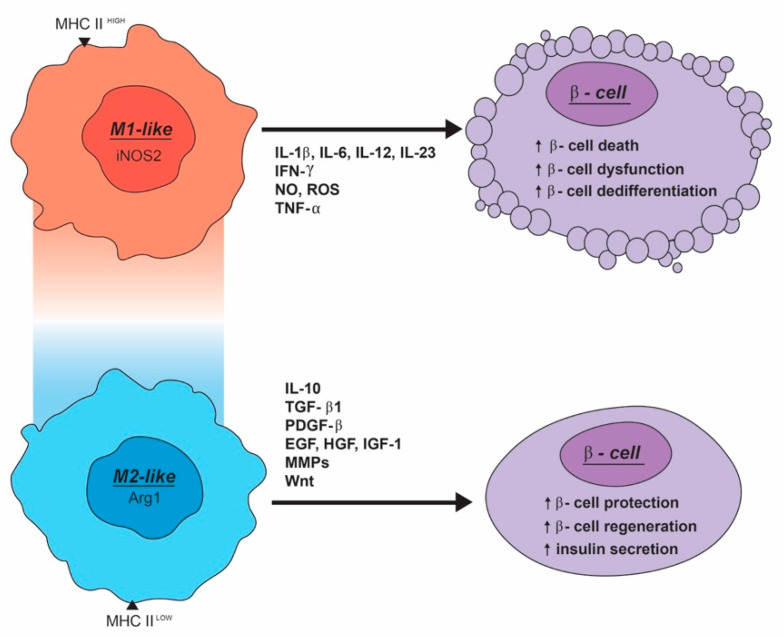
How the M1-like and M2-like extremes of the macrophage activation spectrum affect β-cell function, survival, and proliferation through secreted factors. M1-like macrophages are inflammatory in nature, producing and secreting inflammatory cytokines, NO, and ROS. M2-like macrophages play more of a protective role by inducing β-cell survival and proliferation. M2-like macrophages secrete anti-inflammatory cytokines, signaling peptides, and enzymes involved in tissue remodeling, inducing β-cell proliferation and enhancing insulin secretion.

A clearer understanding of the processes of macrophage activation and the macrophage produced factors that directly affect the β-cell could be utilized as a potential therapeutic to maintain and expand functional β-cell mass as a treatment for both T1D and T2D (Figure 2). Furthermore, understanding the differences between resident and recruited macrophages and their ability to maintain or damage β-cells may be leveraged to improve patient care. In addition to having direct application to T1D, these findings may be applicable to other autoimmune disorders. Similarly, understanding the macrophage-mediated changes observed at the pancreatic islet in T2D may be beneficial for other obesity-related pathologies in other organs.

## Figures and Tables

**Table 1:** Macrophages secreted factors that have a negative effect on functional β-cell mass.

Effectors	Target
IL-1β	Initiates β-cell apoptosis through ERK signaling pathways [18,64] Decreases insulin mRNA levels [57,58,59,60,61,62,63] Impairs GSIS [57,58,59,60,61,62,63] Increases IL-6 release in β-cell [18] Transcriptional changes of 3068 genes associated with inflammation, cell death, antigen presentation, and cytokines/chemokines [65] Contributes to increased ER stress [68] Increases Fas expression [77]
IL-6	Impairs GSIS [76,77] Decreases Ins1, Ins2, and PDX1 mRNA levels in the islet [79]
IFN-γ	Participates with IL-1β to activate NF-κB genes, leading to NO and cytokine production leading to ER stress [65] Participates with IL-1β to cause transcriptional changes to 3068 genes associated with inflammation, cell death, antigen presentation, and cytokines/chemokines [65] Impairs β-cell insulin secretion [84,85]
TNF-α	Contributes to increased ER stress [68] Participates in NF-κB pathway activation [92] Activates proapoptotic and proinflammatory pathways through NF-κB [49,91,93] Increases iNOS and NADPH oxidase activity, leading to increased ROS production and mitochondrial damage [94,95,96,97] Induces intrinsic apoptosis [97] Increases the expression of cytokines CXCL1, CXCL8, CCL20, CCL2, and CXCL10, which promote immune cell infiltration of the islet [100] Induces Ca^2+^ influx in β-cells, impairing insulin secretion [101]

**Table 2:** Macrophages secreted factors that have a positive effect on functional β-cell mass.

Effectors	Target
WNT3A	Increases β-cell proliferation and survival via Wnt/β-catenin pathway [107,117,118]
RETINOIC ACID	Increased expression of RARβ and increased insulin production and secretion [119]
TGFβ1	Induces upregulation of SMAD7, which is responsible for increased β-cell proliferation [111] Induces upregulation of SMAD2, which is a SMAD7 inhibitor [111]
EGF	Inhibits SMAD2 nuclear localization, working in conjunction with TGFβ1 to induce β-cell proliferation [111]
PDGF	Induces β-cell proliferation [122]
IGF-1	Promotes β-cell survival by maintaining GSIS [126]
IL-10	Induces upregulation of anti-apoptotic genes, promoting greater β-cell survival [127] Increases iNOS levels, decreasing NO levels in β-cells [128] Increases insulin secretion [129]
MMP9	Promotes islet vascularization and β-cell expansion [113]

**Table 3:** References that studied the effect of M1-like or M2-like macrophages on rodent islets, human islets, or both.

Rodent or Human Islets	References
Rodent	[18,25,26,31,32,33,34,37,38,40,42,45,46,47,50,51,52,55,56,58,62,64,67,71,73,79,86,89,94,98,99,102,103,106,107,108,111,113,115,116,118,119,121,123,124,127,132,133]
Human	[27,28,30,36,39,43,48,61,63,69,77,78,100,104,117,130,131]
Both	[5,17,20,59,66,76,93,97,120]

## Abbreviations

Aldh1a2	Aldehyde dehydrogenase 1 family, member A2
ARG1	Arginase 1
ATF3	Activating transcription factor 3
ATF4	Activating transcription factor 4
ATP	Adenosine triphosphate
BET	Bromodomain and extraterminal domain family
BIP	Binding immunoglobulin protein
CCL17	Chemokine (C-C motif) ligand 17
CCL20	Chemokine (C-C motif) ligand 20
CCL24	Chemokine (C-C motif) ligand 24
CCL5	Chemokine (C-C motif) ligand 5
CHI3L1	Chitinase 3 Like 1
CHOP	CCAAT/enhancer binding protein (C/EBP) homologous protein
CSF-1	Macrophage colony-stimulating factor
CSF-1R	Colony-stimulating 1 receptor
CSF-2	Granulocyte-macrophage colony-stimulating factor
CXCL1	Chemokine (C-X-C motif) ligand 1
CXCL10	Chemokine (C-X-C motif) ligand 10 (see also IP-10)
CXCL2	Chemokine (C-X-C motif) ligand 2
CXCL8	Chemokine (C-X-C motif) ligand 8
CXCL9	Chemokine (C-X-C motif) ligand 9
CXCR2	Chemokine (C-X-C motif) receptor 2
Db/db	Diabetic mouse model
DNA	Deoxyribonucleic acid
DP-BB	BioBreeding Diabetes Prone rat
ECM	Extracellular matrix
EGF	Endothelial growth factor
ER	Endoplasmic reticulum
ERK	Extracellular signal-regulated kinase
Fas	Apoptosis antigen 1
Foxo1	Forkhead box protein O1
GCSF	Granulocyte colony-stimulating factor
GK	Goto-Kakizaki rat
GSIS	Glucose-stimulated insulin secretion
HGF	Hepatocyte growth factor
I-BET151	BET bromodomain inhibitor
IFN-γ	Interferon-gamma
IFNGR	Interferon gamma receptor
IGF	Insulin-like growth factor
IGF-1	Insulin-like growth factor 1
IL-1	Interleukin-1
IL-12	Interleukin-12
IL-13	Interleukin-13
IL-18	Interleukin-18
IL-1β	Interleukin-1 beta
IL-21	Interleukin-21
IL-33	Interleukin-33
IL-4	Interleukin-4
IL-6	Interleukin-6
IL-8	Interleukin-8
iNOS	Inducible nitric oxide synthase
iNOS2	Inducible nitric oxide synthase 2
IP-10	Chemokine (C-X-C motif) ligand 10 (see also CXCL10)
IRF-1	Interferon regulatory factor 1
Isl-1	Insulin gene enhancer protein 1
JAK/STAT	Janus kinase/signal transducer and activator of transcription
JNK	c-Jun N-terminal kinase
Kcnj11	Potassium Inwardly Rectifying Channel Subfamily J Member 11
LPS	Lipopolysaccharide
mAb	Monoclonal antibody
MafA	MAF BZIP Transcription Factor A
MAPK	Mitogen-activated protein kinase
MCP-1	Monocyte chemoattractant protein-1
M-CSF	Macrophage colony-stimulating factor
MerTK	Tyrosine-protein kinase MER
Min6	Mouse insulinoma cell line
MIP1α	Macrophage inflammatory protein-1 alpha
MMP	Metalloproteinase
MMP9	Metalloproteinase 9
mRNA	Messenger ribonucleic acid
NAD	Nicotinamide adenine dinucleotide
NADPH	Nicotinamide adenine dinucleotide phosphate
NF-κB	Nuclear factor kappa-light-chain-enhancer of activated B cells
NLRP3	NOD-, LRR- and pyrin domain containing 3
NO	Nitric oxide
NOD	Non-obese diabetic
NOS	Nitrogen species
NOS2	Nitric oxide synthase
p27^CDKN1B^	Cyclin-dependent kinase inhibitor 1B
PARP	Poly (ADP-ribose) polymerase
PDGF	Platelet-derived growth factor
PDGFR	Platelet-derived growth factor receptor
PDX1	Pancreatic and duodenal homeobox 1
PI3K-AKT	Phosphatidylinositol 3-kinase-protein kinase B signaling pathway
RARβ	Retinoic acid receptor beta
ROS	Reactive oxygen species
SMAD2	Mothers against decapentaplegic homolog 2
SMAD7	Mothers against decapentaplegic homolog 7
Sox9	SRY-Box Transcription Factor 9
STAT1	Signal transducer and activator of transcription 1
STAT3	Signal transducer and activator of transcription 3
STZ	Streptozotocin
T1D	Type 1 diabetes
T2D	Type 2 diabetes
TGFβ	Transforming growth factor alpha
TGF-β1	Transforming growth factor beta 1
Th1	T-helper type 1 cell
Th2	T-helper type 2 cell
TNF-a	Tumor necrosis factor alpha
TNFR	Tumor necrosis factor receptor
Ucn3	Urocortin-3
VEGF	Vascular endothelial growth factor
VEGF-A	Vascular endothelial growth factor A
Wnt3a	Wingless-Type MMTV Integration Site Family, Member 3A
XBP1	X-box binding protein-1
YM1	Synonym for chitinase-like 3
ZDF	Zucker diabetic fatty rat

## Note

References [5,30,33,37,38,39,43,47,58,69,76,102,103,104,107,113,115,116,117,122,130,131,132,133] indicates key finding in the field, relative to this topic.
